# Supplementary material for: Pandemic news information uncertainty—News dynamics mirror differential response strategies to COVID-19
Source: PLoS One. 2023 Jan 11;18(1):e0278098. doi: 10.1371/journal.pone.0278098 (PMC9833533; doi:10.1371/journal.pone.0278098)
Supplement: Attachment — Submitted filename: Response-to-Reviewers.pdf [file pone.0278098.s001.pdf]

Response to Reviewers

Title: Pandemic news information uncertainty - News dynamics mirror differential response strategies to COVID-19
Authors: K. Nielbo, K. Enevoldsen, R. Baghlini, E. Fano, A.Roepstorff, J.Gao
Contact: Kristoffer Nielbo, kln_at_cas_dot_au_dot_dk

We would like to use the opportunity to thank the reviewers for their valuable comments. We have done our best to address their concerns and improve the manuscript accordingly. Below we will summarize their comments in sub-headings and reference where we have addressed them in the revised manuscript.

The manuscript has been through an additional language review after the revisions.

R1: Generalizability of the results

The short answer is that we do not know how well at the moment this generalizes, but we have added references to studies that point in that direction and discuss the methodological relevance of our approach. We have added additional content regarding the scope of the study, see additional lines: 94-97 , 350-355.

R2: Evidence, confounding factors and variation in start/end points

In general we have made it more explicit that the manuscript is only modeling how newspapers *mirror* two response strategies (scientific vs. scientific & political) and the related behavior (lockdown vs no lockdown), see additional lines: 23-27; 94-97; 101; 105-108; 300-306

This comment has multiple sub-items:

1. factors: political events, international pandemics, newspaper selling, internet resources:
    - The motivation for choosing Sweden and Denmark was exactly that both nations are very similar on most confounding factors. To address the specific concerns we have reference reports in footnote four that show that internet access and media consumption during 2020 were very similar for Denmark and Sweden. Political events are more complicated, but from a qualitative survey of news, we have any major political events during the period that were not directly related to COVID-19 (also mentioned in footnote four).

    - We mention key events in Sweden that co-insides with the Danish lockdown, lines 272-278.

2. start/end variation

    - Regarding the slightly different starting and ending points in Denmark, we have added a explanation essentially about the gradual nature of the COVID-19 news event, see lines 230-244

R2: Clarification of statistical explanations

This comment has multiple sub-items:

1. Novelty-Resonance Baseline
    - For more detail on the baseline, we have now added table 2 that contains slopes and 95% confidence intervals for figure 1.
2. The authors stated Denmark shows a stronger average association than Sweden. However, the values of mean and SD overlapped for the two countries
    - deleted
3. “significant” decreases in novelty for Sweden newspapers were not pointed out.
    - We checked the robustness of the model’s detected ‘valleys’ (as opposed to other observable `valleys’) with a frequentist method for change detection, now mentioned in footnote 5.
    - Lines 196-203 explain in more detail that the model is agnostic to the location of the change points, iow. if ‘the decreases in novelty for Sweden’ were indeed significant changes to the mean, the model will detect them.
    - Additional numerical explanation of the change in newspapers with change points in lines 280-82
4. difference between Fig.1 and Fig.3 should be addressed by mathematical analyses.
    - For analysis of Fig.1 vs Fig.3, we have now added table 2 that contains slopes and 95% confidence intervals for both.
    - Additional explanation about the difference between the figures in lines 272-278.

R2: Provide content examples

While all newspaper content is copyrighted and we are not allowed to share parts in any form (except for reviewers), we have provided a few examples of titles (from a public media database) and an explanation of changes to content in lines 221-230.

R2: Description in the ms is subjective

We have added additional references to recent studies that support the more descriptive parts

- Baekgaard M , Christensen J, Madsen JK , Mikkelsen KS Rallying around the flag in times of COVID-19 Journal of Behavioral Public Administration. 2020;3(2). doi:10.30636/jbpa.32.17
- Brusselaers N, Steadson D, Bjorklund K, Breland S, Stilhoff S¨orensen J, Ewing A, Bergmann S, Steineck G. Evaluation of science advice during the COVID-19 pandemic in Sweden Humanities and Social Sciences Communications. 2022;9(1):1–19. doi:10.1057/s41599-022-01097-5
- Wevers M, Kostkan J, Nielbo KL Event Flow - How Events Shaped the Flow of the News, 1950-1995 Proceedings for the CHR 2021: Computational Humanities Research Conference, November 17–19, 2021.
